# Treatment of Pulmonary Consumption
†The Treatment and Cure of Pulmonary Consumption, on Principles Natural, Rational, Scientific, and Successful. By George Bodington, Surgeon. London: Longmans. 1840.


**Published:** 1857-01

**Authors:** 


### TREATMENT OF PULMONARY CONSUMPTION.†

Although this book was written long ago, it is new to us; and we notice it specially, because it is the express wish of

† The Treatment and Cure of Pulmonary Consumption, on Principles Natural, Rational, Scientific, and Successful. By George Bodington, Surgeon. London: Longmans. 1840.

the editor to say that Mr. Bodington, in this brief treatise, had anticipated many arguments and facts of a similar nature to those given in the paper on the *Hygienic Treatment of Pulmonary Consumption*, in our last number. All these would have been noticed had they been known at the time. It is not too late to note some of them now, nor will the labour be lost. At the time when Mr. Bodington's essay appeared it was rather severely handled, as we are informed, by the critics. It will be satisfactory to him to see that the times have changed, and that a periodical specially devoted to the independent advocacy of such truths as he so boldly set forth, now finds an educated and scientific circle of readers, and is worked by a staff of contributors who are thought all the sounder as practitioners because they go in for essentials, and protest for natural and rational principles in the prevention and treatment of disease.

Speaking of pure air in the treatment of consumption, the author remarks:—

"I believe, having mentioned the shutting up plan in close rooms, the use of antimony and digitalis, if I add the use of demulcents, of blisters, leeches, plasters, etc., I shall have described the helpless and meagre system of medical treatment of consumption in general use at the present day, the utter uselessness of which is so well known and so obvious, that the members of the medical profession in the towns are in the habit of dismissing their patients to some distant sea-port or watering-place, where, falling under precisely the same mode of treatment, they commonly die. . . . . . . . There is nothing gained by resorting to the coast; in truth, the interior of the island is the best; the air is just as pure and much milder, and more suitable for the lungs of consumptive people, if they will but breathe it. There is but one other proposition in the way of treatment to which I have to allude, I mean to the inhalation of gases of various kinds, by which means it is proposed to convert the cough of consumption into a catarrhal cough, which catarrh is to continue so long as the patient lives, or, discontinuing, the consumption would supervene. . . . The only gas fit for the lungs is the pure atmosphere freely administered, without fear; its privation is the most constant and frequent cause of the progress of the disease. To live in and breathe freely the open air, without being deterred by the wind or weather, is one important and essential remedy in arresting its progress;—one about which there appears to have generally prevailed, a groundless alarm lest the consumptive patient should take cold. Thus one of the essential measures necessary for the cure of this fatal disease is neglected, from the fear of suffering or incurring another disease of trifling import. No two diseases can be more distinct from each other than consumption and catarrh; it is the latter only which might be caught by exposure to atmospheric causes; with the former they have nothing to do. Farmers, shepherds, ploughmen, etc., are rarely liable to consumption, living constantly in the open air; whilst the inhabitants of the towns, and persons living much in close rooms, or whose occupations confine them many hours within doors, are its victims: The habits of these latter ought, in the treatment of the disease, to be made to resemble as much as possible those of the former class, as respects air and exercise, in order to effect a cure. How little does the plan of shutting up the patients in close rooms accord with this simple and obvious principle. As to the result of such a practice, it is known to all, one-fifth of the deaths annually in England are from consumption, whilst cures are scarcely ever heard of and never expected.

"The most important remedial agent in the cure of consumption, is that of the free use of a pure atmosphere; not the impure air of a close room, or even that of the house generally, but the air out of doors, early in the morning, either by riding or walking; the latter when the patients are able, but generally they are unable to continue sufficiently long in the open air on foot, therefore riding or carriage exercise should be employed for several hours daily, with intervals of walking as much as the strength will allow of, gradually increasing the length of the walk until it can be maintained easily several hours every day. The abode of the patient should be in an airy house in the country; if on an eminence the better. The neighbourhood chosen should be dry and high; the soil, generally of a light loam, a sandy or gravelly bottom; the atmosphere is in such situations comparatively free from fogs and dampness. The patient ought never to be deterred by the state of the weather from exercise in the open air; if wet and rainy, a covered vehicle should be employed, with open windows. The cold is never too severe for the consumptive patient in this climate; the cooler the air which passes into the lungs, the greater will be the benefit the patient will derive. Sharp frosty days in the winter season are most favourable. The application of cold pure air to the interior surface of the lungs is the most powerful sedative that can be applied, and does more to promote the healing and closing of cavities and ulcers of the lungs than any other means that can be employed. . . . Many persons are alarmed and deterred from taking much exercise in the open air, from the circumstance of their coughing much on their first emerging from the warm room of a house; but this shows that the air of the room was too warm, not that the common atmosphere was too cold. To live in a temperature nearly equal to the latter at all times should be the aim of the patient, who should avoid warm close rooms as much as possible, and always keep away from the fire, taking care to keep the surface of the body warm by sufficient clothing. Thus the equal temperature so much considered, and said to be necessary, should be that of the external air, instead of that so commonly employed, the warmth of a close room.

"The powerful effect of the early morning air, in allaying excitement, is so great and so superior to all other means, that it should, in my opinion, under the eye and by the regulation of the medical attendant, form the foundation of the whole course of treatment; without it, he will not be enabled to administer the due proportion of stimulating and nutritious aliment; it is the proper preparation for the administration of medicinal sedatives; by it the muscular power is preserved from undue exhaustion, and the sanguiferous system from running away in waste; for this course of treatment I have invariably found to diminish the rapidity of the pulse. The profuse nocturnal perspirations are also soon subdued by this method of treatment, and the great debility they occasion avoided. The skin assumes a healthier action in proportion to the extent of exposure to the external atmosphere, particularly to the morning air."

Referring to diet, he observes:—

"In order to restore a consumptive patient, it will be necessary especially to attend to the following matters. We shall find first of all a rapid and weak pulse, ranging from 120 to 140 beats a minute, clearly indicating a deficient supply of blood, and the heart and arteries irritable in proportion to this deficiency. This condition must be met at once, not by the means termed 'antiphlogistic,' but with frequent supplies, in moderate quantities, of nourishing diet and wine; a glass of good sherry or Madeira in the forenoon, with an egg, another glass of wine after dinner, fresh meat for dinner, some nourishing food for supper, such as sago, or boiled milk, according to the taste and digestive powers of the patient. This will be supplying means to rectify the morbid conditions of the nutritive functions, and to allay the irritability of the heart and arteries."

Lastly, concerning hospitals for consumptives, he adds:—

"With respect to the consumptive poor patients, those who cannot afford to pay for a proper treatment of this sort, hospitals should be established in the vicinity of large towns, in fit situations, and properly appointed in all respects for their reception and treatment. In these there should be provision made for affording them carriage or horse exercise; and gardening, and farming occupations, for the convalescent. The common hospital in a large town is the most unfit place imaginable for consumptive patients, and the treatment generally employed there very inefficient, arising from the inadequacy of the means at command. . . . Connected with a consumption hospital, provision should be made for the employment of the convalescent and cured patients, who ought never to return to their former occupation, but should be employed after as agricultural labourers, gardeners, or in any other pursuit, rather than return to their former occupation."

Of the pathological views of Mr. Bodington we do not speak; indeed, the science of pathology has so changed since

1840, that he would probably not now hold out himself in favour of these. The practice he may well cherish, however; for it is becoming more and more widely recognised as the world revolves each day.

